# Perception of Justice and Employees’ Brand-Based Equity in the Service Sector: Evidence From Education Industry

**DOI:** 10.3389/fpsyg.2022.871984

**Published:** 2022-04-15

**Authors:** Lu Li

**Affiliations:** Chinese Opera Institute, Shandong College of Arts, Jinan, China

**Keywords:** employee brand-based quality, perception of justice, brand endorsement, brand allegiance, brand consistent behavior, psychological contract fulfillment

## Abstract

This study aims to investigate the impact of justice perception of the employees on three dimensions of employee-based brand equity (EBBE) under the mediating role of psychological contract fulfillment. For this purpose, data have been collected from the employees of the education industry under the convenience sampling technique. In this regard, a survey method was used, and questionnaires were distributed among 420 respondents, out of which 310 questionnaires were received back, and after discarding 32 partially filled questionnaires, useable responses were left (279 observations). Data have been analyzed through structural equation modeling, and the partial least square (PLS)-SEM approach has been used in this regard through the Smart PLS software. Measurement and structural models were assessed, and all the indicators of reliability and validity have been found to be fit. Path estimation indicates that perception of justice promotes brand endorsement and brand allegiance, while the relationship of perception of justice and brand-consistent behavior has not been found statistically significant. Moreover, it has also been found that perception of justice ensures employees that their psychological contract has been met. In addition, psychological contract fulfillment has found a mediating mechanism between the perception of justice and the three dimensions of EBBE.

## Introduction

Brand equity, or perhaps the value given to a product by the brand, is a major indicator of brand and commercial success and, therefore, is frequently a company’s most valuable asset (Ambler, 2000; Myers, 2003). In contrast, the research acknowledges that this value may be received by a variety of stakeholder groups, the most published scientific studies, and brand equity from customer’s or firm’s perspective (Christodoulides and de Chernatony, 2010; Veloutsou and Guzman, 2017). Employees’ importance in fulfilling any brand promise to various parties (e.g., consumers) is extensively established, especially in the context of services. Employee knowledge and talents, for instance, play a crucial role in the consumers’ brand experience and, as a result, their brand conceptions (de Chernatony and Cottam, 2006). Limited research has been done on how to improve employee-based brand equity (EBBE), such that stakeholders may better execute their responsibilities as endorsers (Helm, 2011; Sirianni et al., 2013; Morokane et al., 2016).

Employees’ perceived added value because of employee-based brand development activities is captured by EBBE (Baumgarth and Schmidt, 2010). Employees’ internalization of the company’s values is a crucial element in internal branding, as continuous execution of the brand promise to consumers is improbable without it (Helm et al., 2016). The success of organizational development depends on internal stakeholders’ synchronization with the company’s values and how this perception equates to brand and/or consumer behaviors (Anbarasan and Sushil, 2018). Customers’ experiences with the brand promise would then remain ineffective without employees’ synchronization with the company’s core values (Helm et al., 2016; Du Preez et al., 2017). Employees’ self-esteem and organizational affiliation are boosted by working for an organization with a good employer brand (Lievens et al., 2007).

By increasing the organization’s trust with workers, consistent implementation of the brand promise sustains strong commitment and high performance among employees and, ultimately, organizational effectiveness (Kashive and Khanna, 2017). Various dimensions of EBBE have been explored in the past but very less attention has been given to the three-dimensional benefits of EBBE, including brand endorsement, brand allegiance, and brand-consistent behavior of employees (Kashive and Khanna, 2017). These dimensions provide core values to branding concepts and should be evaluated for developing brand equity among employees. This is based on external employee marketing/conveyance of the brand to everyone else, or referral, which is seen as a significant part of brand promoting conducts (Shinnar et al., 2004). The willingness of an employee to speak positively about the company (brand) as well as to quickly suggest the company (brand) to anyone is referred to as brand endorsement (Piehler, 2018).

Few studies have supported the notion that workers who seem to have a favorable attitude about their employers are organically driven to engage in meaningful information exchange. Employee involvement has several advantages. Interpersonal advocacy by employees sometimes leads to beneficial organizational productivity, such as reduced recruiting costs, improved job performance, and enhanced pre-employment awareness, all of which influence organizational integration. Employee brand allegiance is related to employees’ future desire to stay with the company (brand). Despite the substantial economic impact of losing competent individuals, this desire is seen as a big choice (Ramlall, 2004). This also aids in the development of important human capital, wherein people are deemed to have skills, expertise, and understanding, which provides economic value for businesses through higher efficiency (Snell and Dean, 1992).

The success of a service brand is exacerbated by the recruitment of efficient personnel who regularly display brand-related activities. This happens because the brand equity claim is continuously executed in an expense-efficient manner. According to studies, an employee’s declaration of the desire to remain employed reflects their understanding of the importance of upholding the brand’s values (Punjaisri and Wilson, 2007). This idea of planned behavior, which claims that the strongest indicator of future actions is the desire to act, exemplifies this future-oriented thinking (Schiffman and Tuncay, 2001). Workers who are comfortable with the workplace environment are more likely to engage in behaviors that go beyond the job’s technically defined standards (Beckett-Camarata, 2003). Brand-consistent behavior, e.g., might be described as an employee’s non-prescribed behavior that is consistent with the organization’s brand values (Burmann et al., 2009).

Another important aspect of product behavior is its voluntary nature, but it is critical for organizational efficiency (Martín-de Castro et al., 2011). This evolves because service organizations are unable to foresee all of the right employee behaviors needed for organizational performance (Deluga, 1994). Burmann and Zeplin (2005) defined brand-consistent behavior, or brand citizenship behavior, as “the crucial (behavioral) ingredient for effective internal brand management.” There are minor variations between brand-related behavior and organizational-related behavior. Equality and fairness in the distribution of appropriate resources, as well as equal and respectful behavior, are social necessities for persons in every circle of acquaintances (Dirks and Ferrin, 2001). A professional social circle is formed by the working environment. Organizational justice has been declared to be one of the most important aspects of organizational practices.

The concept of justice may be defined as people’s and worker’ perceptions of fair and equal treatment. Distributive justice, procedural justice, and interactional justice are the three forms of organizational justice that have been examined (De Cremer, 2005; Chandio et al., 2020). When it comes to resource allocation and distribution justice, an individual is accustomed to compare his or her rewards with those of his or her counterparts and those of people in comparable positions in other organizations; at the very least, he or she wants to be in balance (Greenberg, 1987). Whereas favorable responses to inquiries on the application of processes on him/her and his/her peers are viewed as fairness in procedure execution, negative responses are perceived as partiality. Employees, as members of the professional community, have a right to expect fairness in allocation and processes, as well as to be treated with dignity.

People in the workplace have been shown to compromise on resource distribution and processes but not on interactional justice (Chandio et al., 2020). Interactional justice is a branch of procedural justice that deals with the human aspect of organizational activities, i.e., how management (or those in charge of rewards and resources) treats the victim of injustice (Ahmad, 2018). In recent times, the importance of how a firm treats its employees has experienced an influx of academics. For example, Patterson suggested that businesses be forced to offer a level playing field for all of their employees (Patterson, 2010). Therefore, based on the gap in evaluation of justice perception on EBBE, this framework of research was designed in which justice perception was taken as an independent variable influencing the EBBE.

The psychological contract as a good idea for describing the existing job interactions has a vast literature (Guest, 2004). A psychological contract is described as “individual views about the parameters of an exchange agreement between individuals and their organization, created by the organization.” In a customized labor market where workers tend to have more tailored relationships with their employers, studying the employment relationship *via* the perspective of the psychological contract is probably well suited. Nevertheless, almost as much as the contents of the agreement are signed directly, the capacity to execute on what has been promised is a critical factor in understanding individual workplace reactions. Organizational culture, organizational citizenship, and turnover intentions are all traditional outcomes connected to psychological contract fulfillment (or lack thereof) (Turnley et al., 2003; Griffin and Sutton, 2004; Sturges et al., 2005).

A few researchers have already explored the mediating impact of psychological contract fulfillment between some factors affecting EBBE (e.g., Deepa and Baral, 2021) but did not evaluate the impact of justice perception on EBBE with psychological contract fulfillment as a helping tool leaving a gap for us to evaluate its mediating link between both. Connecting the ties between perceptions of justice with EBBE, this study focused on evaluating the relationship between justice perception and brand endorsement, brand allegiance, and brand-consistent behaviors. This study also provided information about a mediating role of psychological contract fulfillment between justice perception and EBBE beneficial dimensions, i.e., brand endorsement, brand allegiance, and brand-consistent behaviors.

## Theoretical Framework and Hypothesis Development

This research is focused on assessing the impact of justice perception on EBBE benefits, such as brand endorsement, brand allegiance, and brand-consistent behaviors, with the mediation aid of psychological contract fulfillment. Most of the research in the past has shown that these attributes are controlled and paved through different theories, such as social exchange theory (SET), social comparison theory, equity theory, psychological contract theory, signaling theory, and affective events theory (Guerrero and Herrbach, 2008; Badawy and El-Fekey, 2017; Bandyopadhyay and Srivastava, 2021).

In management studies, the signaling theory may make you realize how companies attempt to sway the attitude of their employees. Employees are expected to behave positively by their employers, and their signals serve to elicit this behavior. As a result, signaling theory emphasizes the deliberate delivery of positive words to diverse workers regarding desired organizational characteristics (Connelly et al., 2010). Therefore, the theory could have an impact on the psychological contract fulfillment of the employees.

Perception of justice and fairness have largely been researched in connection with productivity using SET, which looks at different types of human transactions. According to theory, any human interaction is founded on subjective cost–benefit analysis (Aryee et al., 2002). According to the equity theory, employees’ perceptions of unjust labor reward distribution cause stress, which they endeavor to rectify.

In contrast, workers want to limit their losses, thus, they don’t want to lower their in-role productivity because it is directly related to their incentives, and therefore, they tend to employ contextual performance as a reaction to fair/unfair reward distribution (Saleem and Gopinath, 2015). The notion of social comparisons was used to provide a new method. Several essential aspects of the organizational setting, such as organizational justice, performance evaluation, and emotional behavior in the workplace, have been explained using social comparison processes by a few scholars (Spence et al., 2011; Saleem and Gopinath, 2015).

Researchers contend that the SET influenced most of the models used to describe organizational justice and employee behavior. While this theory brought a lot to our comprehension, it still does not describe why workers participate in such behaviors (Goodman and Haisley, 2007; Greenberg et al., 2007; Spence et al., 2011). Festinger established the principle of social comparison as a source of knowledge for self-evaluation in 1954. According to the study by Festinger, the social comparison would be a normal and fundamental human need in which individuals compare their thoughts and talents with those of others, particularly when objective physical criteria are lacking. A practice of pondering about facts around one or more individuals’ tendencies and opinions in connection to one’s ability and opinion is referred to as social comparison (Festinger, 1954; Goodman and Haisley, 2007). The roots of organizational justice and social comparison are evident since justice theory is originally based on the Adams’ equity theory (Adams, 1965), which was inspired by the social comparisons theory (Festinger, 1954).

These views are based on the same hypothesis, which is that people compare things. People compare their inputs and outputs with some referent others who are primarily at the same level to assess the fairness of their outcomes in equity theory, whereas in the social comparisons theory, individuals compare their ability and opinions with others after rating them in different levels (above or below) for self-evaluation, consciousness, and identity (Kim et al., 2015). Social similarities exist in two different directions, namely, upwardly with reference groups who are rated higher than the assessor level, and downwardly with reference groups who are rated lower than the evaluator level (Van Yperen et al., 2006). Individuals’ perceptions of their talents and attitudes are formed as a result of this comparison (Moon et al., 2016). This impression can be either favorable or negative, resulting in a positive or negative emotional state. Individuals’ responses toward perceived justice are influenced by their emotional state (Collie et al., 2002). These theories provided a basis for assessing the relationships of this study.

### Association of Perception of Justice With Employee-Based Brand Equity

Justice is a basic principle in businesses, and whether there’s a promotion determination, work assignment, incentive distribution, or another sort of social transaction, issues of fairness will always emerge (Konovsky and Cropanzano, 1991). In the literature, the concepts “justice,” “fairness,” and “equity” have all been used interchangeably. Any event, activity, or decision is assessed as fair or unfair depending on the individual’s views about the choice and his or her value or normative system in relation to all those beliefs. Humans are social beings, and businesses must develop environments that allow people to connect socially (Moorman, 1991; Bies, 2005; Coetzee, 2006; Suliman and Al Kathairi, 2013). Therefore, many types of activities that happen between employees at work have been investigated in the literature. In complaint and service recovery situations, justice theory gives a platform for understanding contentment (Allen et al., 2014).

Distributive, procedural, and interactional justice are the three dimensions of justice. A distributive element is concerned with the perceived justice of results, whereas the procedural element is concerned with the justice of the rules and methods used to generate the consequence. The interpersonal care individuals receive during the dispute resolution mechanism that is the emphasis of the interactional element. Many facets of a service provider’s operations are likely to have an impact on people’s perceptions of interactional fairness (Hazée et al., 2017). Because judgments of the outcome’s justice are more heavily influenced by human emotions, procedural justice is less visible (Zhao et al., 2012). Customers may find it difficult to discern between procedural and interactional justice because many features of both are linked. As a result, for service firms, interactional fairness tends to play a larger role in determining happiness. For instance, whether or not such a store allows an unhappy consumer to describe the situation whether or not the merchant considers that knowledge is both critical components of grievance interaction (Lee et al., 2017).

Customers are less worried about procedural fairness when they have more clear and explicit information about the service provider’s reliability, according to a few studies. Whenever customers believe the supplier can be entrusted to follow fair procedures, they are more sensitive to the fairness of the conclusion. All interactional and distributive fairness have a considerable influence on perceived satisfaction, while procedural justice does not (Akram et al., 2017). Few studies support the notion that workers who seem to have a favorable attitude about their employers are organically driven to engage in meaningful information exchange. Employee involvement has several advantages. Interpersonal advocacy by employees sometimes leads to beneficial organizational productivity, such as reduced recruiting costs, improved job performance, and enhanced pre-employment awareness, all of which influence organizational integration.

Employee brand allegiance is related to employees’ future desire to stay with the company (brand). Despite the substantial economic impact of losing competent individuals, this desire is seen as a big choice (Ramlall, 2004). This also aids in the development of important human capital, wherein people are deemed to have skills, expertise, and understanding, which provides economic value for businesses through higher efficiency (Snell and Dean, 1992). Workers who are comfortable with the workplace environment are more likely to engage in behaviors that go beyond the job’s technically defined standards (Beckett-Camarata, 2003).

For example, brand-consistent behavior might be described as an employee’s non-prescribed behavior that is consistent with the organization’s brand values (Burmann et al., 2009). Burmann et al. (2009) defined the brand-consistent behavior, or brand citizenship behavior, as “the crucial (behavioral) ingredient for effective internal brand management”. There are minor variations between brand-related behavior and organizational-related behavior (Burmann and Zeplin, 2005). It is assumed like this because brands are developed through organizations, and the behaviors of employees toward their organizations lead them to develop the same behaviors for the brand (Brethower et al., 2021).

**FIGURE 1:**
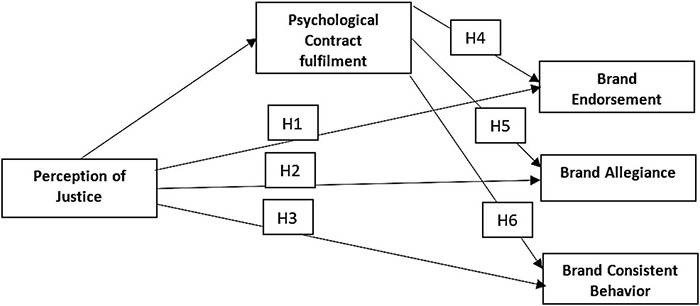
Conceptual framework.

A lot of consideration in the past has been given to different aspects of customer-based brand equity but very less attention has been given to EBBE so far. The major exploration of perceived organizational justice remained unanswered in the past toward creating EBBE. Therefore, we designed this research, and, in this regard, we proposed the following hypotheses to evaluate the impact of the perception of justice of employees toward creating EBBE (see Figure 1 for conceptual framework).

**H1**. Perception of justice is associated with brand endorsement.

**H2**. Perception of justice is associated with brand allegiance.

**H3**. Perception of justice is associated with brand-consistent behavior.

### Mediating Role of Psychological Contract Fulfillment

Levinson was the first to discuss the psychological contract in full in Levinson et al. (1962). Mutual duties exist between the employer and the employee, some of which are explicitly established in the job’s terms and conditions and the employment contract. Expectations concerning job stability, work content, career growth, and less well-defined incentives and perks are also less formal. These expectations make up the psychological contract. Certain aspirations, including salary, vacation pay, and sick pay, are clearly stated and recognized, while others, such as job stability and promotion chances, are simply hinted at.

The psychological contract is founded on reciprocity (it binds both parties) and proportionality (it entails an equal interchange among both stakeholders), as well as the idea of organizational justice. If the company refuses and is unable to fulfill the contract, it may have negative effects on both the individual and the company. As the psychological contract is not well defined or officially recognized, the employer and employee may have quite different ideas about what constitutes a violation (Nimmo, 2018). Both mutual trust and reciprocity are linked to increased performance and progress in the workplace. They are also linked to self-reported indicators of met expectations and intend to work for the same company in the future.

Mutual adherence to the psychological contract does have a favorable impact on the workplace. A variety of harmful behaviors are linked to a violation of the psychological contract. Psychological contract violation was found to lower organizational trust, have fewer collaborative workplace conditions, and have increased absenteeism in a sample of customer service personnel. Employees may regard inconsistencies between an organization’s declared behavioral norms and its actual behavior as a contract breach (Dabos and Rousseau, 2004; Deery et al., 2006).

Several researchers in the past have looked into the relationship of perceived justice and psychological contract fulfillment in which scientists explored the impact of psychological contract fulfillment on organizational justice (Estreder et al., 2019). Psychological contracts are characterized by mutual interaction of perceived commitments and responsibilities that describe the criteria of the workforce relationship between the employer (i.e., inducements, namely, participation in decision-making, working conditions, and employment security) and the employee (i.e., contributions, namely, protecting the company’s image, accepting an internal transfer if necessary, and working overtime or extra hours when required).

The degree to which employees perform psychological contract fulfillment or violation is an important factor to consider when assessing their job relationship. Failing to maintain the commitments made in the psychological contract results in negative work outcomes, such as lower job satisfaction, organizational commitment, in-role competence, and organizational citizenship behaviors, as well as greater desire to quit or attrition (Dabos and Rousseau, 2004; Guest, 2004). Psychological contract fulfillment has given some promising and significant results in some past studies as a mediator (Ashfaq et al., 2018; Saeed, 2020; Bandyopadhyay and Srivastava, 2021; Gulzar et al., 2021). Therefore, we hypothesized that psychological contract fulfillment could also mediate between justice perception and benefits of EBBE, such as brand endorsement, brand allegiance, and brand-consistent behaviors of employees (see Figure 1 for conceptual framework). The hypotheses are as follows.

**H4**. Psychological contract fulfillment mediates the relationship between perception of justice and brand endorsement.

**H5**. Psychological contract fulfillment mediates the relationship between perception of justice and brand allegiance.

**H6**. Psychological contract fulfillment mediates the relationship between perception of justice and brand-consistent behavior.

## Methods

### Participants and Procedure

Participants in this study were approached through a cross-sectional research design keeping in view the theoretical orientation of the study. A non-probability sampling technique was followed, and data were obtained through the convenience sampling method. This technique provides ease and access to approach respondents with low time and low-cost benefits. Moreover, this technique was most suitable, as the respondents were from the service sector (i.e., education sector) where the nature of jobs and activities performed by the employees remains almost symmetrical. For this purpose, a sample of 420 respondents was approached in this regard to meet a reasonable and suitable sample size. This sample size was representative of population based on several criteria; for instance, as a general rule of thumb, we need five to ten respondents against each study construct. In contrast, in this study, a total of five study constructs have been operationalized, so a sample of 50 respondents was justified.

Moreover, according to the criteria recommended by Krejcie and Morgan (1970), in a cross-sectional study design, a total of 384 is sufficient. In this regard, 310 questionnaires were received back, and after eliminating the missing and incomplete responses, the sample size left to 278 observations which have been used in the final data analysis. Thus, this study met the sample size requirement sufficiently. Before administrating the questionnaires on large scale, prior permission from the concerned institution head was obtained. Respondents were briefed about the study purpose, nature of the study constructs being investigated, and their informed consent was obtained. Moreover, they were assured about the data anonymity and confidentiality issues. Thus, this technique has helped us to reduce the error in estimation as a result of common method biases (Ng and Feldman, 2013).

As noted in earlier studies that first-line employees are occupied extensively in their duties and it is a chance that they might face time difficulties in reporting the survey items, we used reverse coded items in the questionnaire to restrict the respondents from providing monotonic responses. This technique also helped us to minimize the common method biases (Malhotra et al., 2006). In addition to this, we placed the study constructs at different places in the questionnaire so that respondents could not generate a correlation. Demographic information of the respondents was also obtained. In this regard, male and female participants constitute almost equal parts of the sample, i.e., 56% of male respondents and 44% of female respondents. The majority of the respondents have an age of fewer than 40 years, while only 20% of respondents have an age more than 40 years. Similarly, most of the respondents were married, while a portion of the respondents living single and as a single parent was 30%.

### Measures

The independent variable of this study (perception of justice) has been operationalized based on 10 items used by Ghosh et al. (2014). This construct was operationalized based on three dimensions, namely, procedural justice, distribute justice, and interactional justice. Sample items for this scale include, “Have you been able to express your views and feelings during those procedures?” and “Does your supervisor communicate details in a timely manner? The original version of this scale contains 20 items, while keeping in view the theoretical orientation of the study, we have used the most relevant items in this study. Similarly, mediating variable of this study, i.e., psychological contract fulfillment is operationalized based on 12 items scale, developed by Guerrero and Herrbach (2008). Four dimensions were covered by this scale, namely career perspective, job content, relationship with others, and compensation. Originally, this scale covers 15 items, whereas this study conceptualized 12 items and three items for each dimension have been used.

Similarly, the scale for EBBE has been used to measure the three dimensions, namely, brand endorsement, brand-consistent behavior, and brand allegiance. Sample items for these dimensions include, “I say positive things about the organization (brand) I work for, “I plan to be with the organization (brand) I work for, for a while,” and “I demonstrate behaviors that are consistent with the brand promise of the organization I work for.” This scale has previously been used in the literature (King and Grace, 2010; King et al., 2012). The reason to use shortened items/scales is based on the recommendation of Hagtvet and Sipos (2016) to reduce the burden on respondents to work through lengthy complete forms. Moreover, during recent years, the trend to create short forms for construct measures is also increasing.

## Data Analysis and Results

This study followed a multivariate data analysis model keeping in view the complex nature of study constructs. For this purpose, a structural modeling approach based on partial least square (PLS)-SEM was used, as recommended by Sarstedt et al. (2014). In this scenario, this technique was most suited because theory related to EBBE is under development, and this study was aimed to explain variance in EBBE based on the perception of justice and fulfillment of the psychological contract (Hair et al., 2017).

As recommended by Hair et al. (2017), structural equation modeling should be assessed based on the measurement and structural model. In this regard, the first measurement model was assessed based on reliability and validity. Thus, the first portion of the analysis is related to the assessment of the measurement model which is related to reliability and validity. Initially, the outer loadings of the measurement model were assessed and items with poor outer loadings were dropped from the analysis. In this regard, two items from the construct perception of justice have been dropped due to poor outer loadings. In this regard, a benchmark of 0.708 value for outer loadings was set. Thus, two items from the perception of justice were with weak loadings, and they were dropped. These two items were PJ-7 and PJ-09. Similarly, two items from psychological contract fulfillment were dropped due to poor outer loadings, namely, PF-09 and PF-11.

No item from the construct EBBE was dropped (see Table 1). However, some items were observed with lower outer loadings values, but these were contributing significantly to the model average of variance extracted (AVE) (i.e., AVE was greater than 0.50, sharing more than 50% of the variance in the construct). From the perspective of reliability and validity (see Table 2), all the constructs indicated strong reliability, and it was observed that all constructs have reliability values greater than 0.60, in terms of alpha, composite reliability, and rho-A (see Table 2). Thus, all the indicators have been found statistically fit, indicating a satisfactory level (values larger than the threshold, i.e., >0.60) (Mela and Kopalle, 2002).

**TABLE 1:** Outer loadings.

Indicator	Brand Allegiance	Brand Consistent Behavior	Brand Endorsement	Perception of Justice	Psychological Contract Fulfillment
BA1	0.815				
BA2	0.613				
BA3	0.748				
BA4	0.720				
BCB1		0.889			
BCB2		0.941			
BCB3		0.894			
BE1			0.800		
BE2			0.834		
BE3			0.808		
BE4			0.811		
PF1					0.815
PF10					0.789
PF12					0.764
PF2					0.892
PF3					0.809
PF4					0.659
PF5					0.896
PF6					0.742
PF7					0.622
PF8					0.813
PJ1				0.646	
PJ10				0.757	
PJ2				0.770	
PJ3				0.734	
PJ4				0.871	
PJ5				0.678	
PJ6				0.823	
PJ8				0.696	

**TABLE 2:** Reliability and validity.

Construct	Cronbach’s Alpha	rho_A	Composite Reliability	Average Variance Extracted (AVE)
Brand Allegiance	0.739	0.823	0.817	0.530
Brand Consistent Behavior	0.893	0.894	0.934	0.825
Brand Endorsement	0.834	0.855	0.886	0.661
Perception of Justice	0.888	0.900	0.911	0.563
Psychological Contract Fulfillment	0.929	0.942	0.941	0.616

As recommended by Fornell and Larcker (1981), discriminant validity was assessed based on the square root of AVE concerning the correlations of the respective construct (Hair et al., 2017). Thus, based on the square root of the AVE of all study constructs, it was observed that the square root of the AVE of all constructs was greater than the correlation value in each row and column (see Table 3; Hair et al., 2011).

**TABLE 3:** Discriminant validity (Fornell–Larker criteria).

Construct	Brand Allegiance	Brand Consistent Behavior	Brand Endorsement	Perception of Justice	Psychological Contract Fulfillment
Brand Allegiance	***0.728***				
Brand Consistent Behavior	0.374	***0.908***			
Brand Endorsement	0.467	0.153	***0.813***		
Perception of Justice	0.511	0.239	0.424	***0.750***	
Psychological Contract Fulfillment	0.733	0.365	0.314	0.314	***0.785***

*Square root of AVE of respective construct is reported in diagonal values.*

**FIGURE 2:**
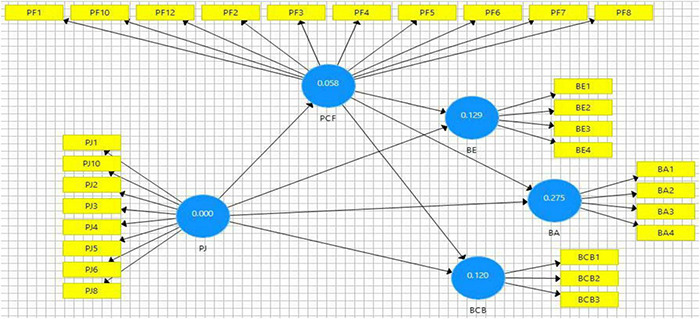
R^2^ depiction.

To assess discriminant validity, we have also employed the heterotrait-monotrait ratio of correlation (HTMT) criteria (see Table 4); here, it has been observed that all the ratios among study constructs have been found less than 0.90. For HTMT, two criteria are followed: one is liberal and the other is conservative; for conservative criteria, the value of the HTMT ratio should be less than 0.85, while in the case of liberal criteria, the value of HTMT should be less than 0.90. This study met both of these threshold limits (see Table 4), namely, brand-consistent behavior and brand endorsement, respectively. Overall model fitness was assessed based on F2, and a satisfactory level of effect size has been observed (see Tables 5, 6; Hair et al., 2017). In addition to this, we have also tested predictive relevance that was assessed based on Q2 and was observed greater than zero (see Figure 2), indicating a good predictive relevance of the model (Geisser, 1975).

**TABLE 4:** Discriminant validity (HTM ratio).

Construct	Brand Allegiance	Brand Consistent Behavior	Brand Endorsement	Perception of Justice	Psychological Contract Fulfillment
Brand Allegiance	-				
Brand Consistent Behavior	**0.419**	-			
Brand Endorsement	**0.563**	**0.171**	-		
Perception of Justice	**0.622**	**0.257**	**0.474**	-	
Psychological Contract Fulfillment	**0.701**	**0.397**	**0.341**	**0.328**	-

**TABLE 5:** R^2^ and adjusted R^2^.

Construct	R Square	R Square Adjusted
Brand Allegiance	0.625	0.622
Brand Consistent Behavior	0.150	0.144
Brand Endorsement	0.216	0.210
Psychological Contract Fulfillment	0.099	0.095

**TABLE 6:** Effect size.

Construct	Brand Allegiance	Brand Consistent Behavior	Brand Endorsement	Perception of Justice	Psychological Contract Fulfillment
Brand Allegiance	-	-	-	-	-
Brand Consistent Behavior	-	-	-	-	-
Brand Endorsement	-	-	-	-	-
Perception of Justice	0.232	0.020	0.150	-	0.109
Psychological Contract Fulfillment	0.972	0.109	0.046	-	-

**FIGURE 3:**
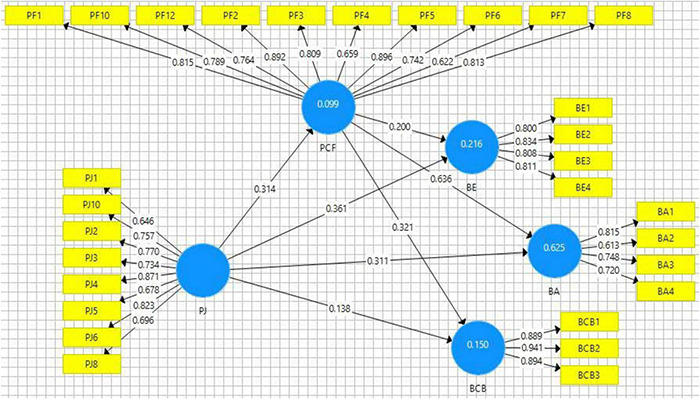
Path estimates.

**FIGURE 4:**
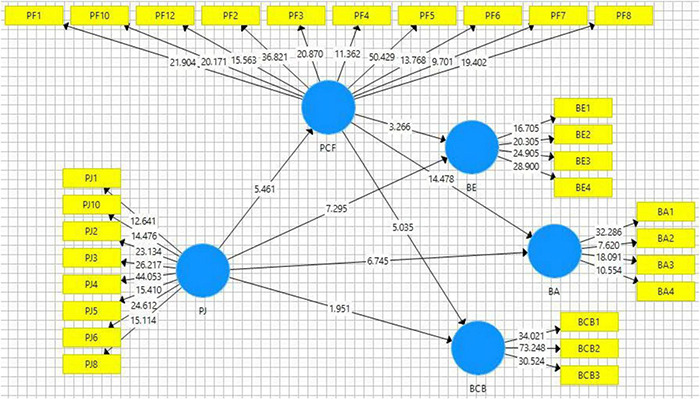
Path significance.

Hypothesis testing has been done based on *t* and *p* statistics (see Figure 3 and Table 7), whereas, in this study, mediation was checked based on indirect path significance. The first hypothesis of this study was based on the relationship of perception of justice and brand endorsement. The values of *t* and *p* were within the acceptable range (*t* = 7.295 and *p* < 0.05) and indicate that this hypothesis is supported (see Table 8); thus, H1 is supported. Empirical findings of this study indicate that perception of justice promotes employees’ sense, and employees perceive that their employer is sincere with them and thus positive feelings are generated among employees. These positive feelings drive employees to advocate positively regarding the employer and they tend to endorse their organization among others. Similarly, the second hypothesis of this study that is related to the perception of justice and brand allegiance was also found statistically significant (*t* = 6.745 and *p* < 0.05), and H2 was supported based on empirical evidence (see Figure 4).

**TABLE 7:** Path estimation.

Direct Paths				
**Path**	**Beta**	**SD**	**t**	**p**
Perception of Justice - > Brand Allegiance	0.312	0.046	6.745	0.000
Perception of Justice - > Brand Consistent Behavior	0.139	0.071	1.951	0.051
Perception of Justice - > Brand Endorsement	0.365	0.050	7.295	0.000
Perception of Justice - > Psychological Contract Fulfillment	0.318	0.058	5.461	0.000
Psychological Contract Fulfillment - > Brand Allegiance	0.639	0.044	14.478	0.000
Psychological Contract Fulfillment - > Brand Consistent Behavior	0.321	0.064	5.035	0.000
Psychological Contract Fulfillment - > Brand Endorsement	0.199	0.061	3.266	0.001

**Indirect Path**	**Beta**	**SD**	**t**	**p**

Perception of Justice - > Psychological Contract Fulfillment - > Brand Consistent Behavior	0.103	0.029	3.445	0.001
Perception of Justice - > Psychological Contract Fulfillment - > Brand Endorsement	0.064	0.025	2.564	0.010
Perception of Justice - > Psychological Contract Fulfillment - > Brand Allegiance	0.202	0.035	5.753	0.000

**Total Paths**				

**Path**	**Beta**	**SD**	**t**	**p**

Perception of Justice - > Brand Allegiance	0.515	0.051	9.955	0.000
Perception of Justice - > Brand Consistent Behavior	0.241	0.070	3.427	0.001
Perception of Justice - > Brand Endorsement	0.429	0.049	8.574	0.000

**TABLE 8:** Hypotheses testing.

		Coefficient (Beta)	S.D	*t*	*p*	Status
**Hypotheses**						
H1	Perception of Justice - > Brand Endorsement	0.365	0.050	7.295	0.000	Supported
H2	Perception of Justice - > Brand Allegiance	0.312	0.046	6.745	0.000	Supported
H3	Perception of Justice - > Brand Consistent Behavior	0.139	0.071	1.951	0.051	Not Supported
**Mediation Hypotheses**						
H4	Perception of Justice - > Psychological Contract Fulfillment - > Brand Endorsement	0.064	0.025	2.564	0.010	Not Supported
H5	Perception of Justice - > Psychological Contract Fulfillment - > Brand Allegiance	0.202	0.035	5.753	0.000	Supported
H6	Perception of Justice - > Psychological Contract Fulfillment - > Brand Consistent Behavior	0.103	0.029	3.445	0.001	Supported

In contrast, H3 of this study related to the relationship of perception of justice and brand-consistent behavior has not been found statistically significant, and H3 is not supported because the values of *t* and *p* were not within an acceptable range (*t* = 1.951 and *p* > 0.05). Moreover, in the case of mediation analysis, indirect paths for H4, H5, and H6 have been found statistically significant which indicates that psychological fulfillment partially mediates the relationship between perception of justice and three dimensions of EBBE. However, this study also employed variance account for (VAF) approach to confirm the nature of the mediation, i.e., either partial or full mediation. For this purpose, an indirect effect of each path was divided through the total effect. In this regard, VAF about the H4 was less than 20% (i.e., 15%) which indicates no mediation, while for the other two paths (i.e., H5 and H6), the value of VAF has been observed as 39% and 43%. Hence, H5 and H6 are supported through the results, while H4 was not found statistically significant.

These findings are supported by the social exchange theory (Blau, 1964). These findings are in alignment with the previous findings that perception of justice has the potency to promote positive behaviors among employees (Sekiguchi, 2007; Bharadwaj, 2014; Erkmen, 2018). Moreover, these findings are in line with the recommendations of McLean Parks et al. (1998) that psychological contract fulfillment is “the idiosyncratic set of reciprocal expectations held by employees concerning their obligations (what they will do for the employer) and their entitlements (what they expect to receive in return).”

Thus, it can be argued that fulfillment of the psychological contract is rooted in the exchange of socioeconomic benefits (Blau, 1964) which comes under the premise of SET (Blau, 1964). In addition, it would be due to the fact that most of the individuals tend to pursue permanent employment as compared with temporary employment (Aronsson and Göransson, 1999), so it might be the reason that there were the employees as most permanent employees. Previous studies have indicated that psychological contract fulfillment results in positive outcomes, such as organizational commitment (Sturges et al., 2005), so findings of these studies are consistent with these studies as the fulfillment of psychological contract can result in EBBE (Morrison and Robinson, 1997; Robinson and Morrison, 2000).

## Discussion

This research was based on identifying the impact or association of perception of justice among the employees of the service section with EBBE benefits, such as brand endorsement, brand allegiance, and brand-consistent behaviors of the employees. The topic of workplace justice has carved out a prominent position in the literature. According to several studies, a greater employee sense of justice has a favorable influence on several facets of EBBE (Ghosh et al., 2014). Justice is a basic principle in businesses, and when there is a promotion determination, work assignment, incentive distribution, or other sorts of social transaction, issues of fairness will always emerge (Konovsky and Cropanzano, 1991). Humans are social beings, and businesses must develop environments that allow people to connect socially (Moorman, 1991; Bies, 2005; Coetzee, 2006; Suliman and Al Kathairi, 2013).

The results of this study produced some very promising insights about justice perception and indicated that a sense of justice among the employees of an organization could develop certain behaviors and attitudes which could lead to EBBE which is also an aim of the employer. They could perform better and develop the brand positively. For instance, the first hypothesis of the study indicated that if the perception of justice prevails between employer and employee, then it could lead to the development of the endorser aptitude in the employee. He could be a good endorser, which would certainly improve brand equity. Researchers in the past did not notice such relationships; hence, this study could be a noble contributor in devising the employer and employee relationship based on justice. The other direct relationships of perception of justice among the employers and employees also produced some promising results and indicated that employees’ perception of justice could lead to brand allegiance or brand loyalty.

The employees will not have any trouble while developing loyalty to their brands. The last direct hypothesis was about the perception of justice with brand-consistent behavior which was proved to be insignificant as, psychologically, employees did not feel secure about promoting the brand. Workers who are comfortable with the workplace environment are more likely to engage in behaviors that go beyond the job’s technically defined standards (Beckett-Camarata, 2003). For example, brand-consistent behavior might be described as an employee’s non-prescribed behavior that is consistent with the organization’s brand values (Burmann et al., 2009). There could be some other factors as well, such as fluctuations in the behaviors of the employers, their behaviors with colleagues, and many others. Previously, this aspect was also neglected in the research.

The indirect effects of psychological contract fulfillment were also studied in this research. This indicates that psychological fulfillment mediates the relationship between perception of justice and three dimensions of EBBE benefits among employees. These findings are supported by the SET (Blau, 1964). These findings are in alignment with the previous findings that perception of justice has the potency to promote positive behaviors among employees (Bharadwaj, 2014; Erkmen, 2018). The indirect effects proved that psychological contract fulfillment could aid in the relationships of perception of justice and EBBE benefits, such as brand endorsement, brand allegiance, and brand-consistent behaviors of employees. The results also proved that the direct effects of perception of justice on brand-consistent behaviors could be rectified with the help of psychological contract fulfillment, indicating that if the contract is fulfilled along with the true justice by the employer, then this kind of relationship could be developed.

These results were in accordance with some previous research in which psychological contract fulfillment positively mediated the relationships in different contexts. Failing to maintain the commitments made in the psychological contract results in negative work outcomes, such as lower job satisfaction, organizational commitment, in-role competence, and organizational citizenship behaviors, as well as greater desire to quit or attrition (Dabos and Rousseau, 2004; Guest, 2004). Psychological contract fulfillment has given some promising and significant results in some past studies as a mediator (Ashfaq et al., 2018; Saeed, 2020; Bandyopadhyay and Srivastava, 2021; Gulzar et al., 2021).

### Theoretical and Practical Contribution

From a theoretical perspective, this study tends to add important theoretical strings to the body of knowledge by exploring the impact of the perception of justice in the service industry. This study includes that perception of justice promotes EBBE and employees tend to advocate their organization when they perceive that their organization is dealing with them fairly. Similarly, this study finds that it is not necessary that employees show their behavior in terms of brand-consistent behavior when they perceive that their firm is dealing with them with justice (De Cuyper et al., 2009). Another contribution of this study is related to the mediating role of psychological contract fulfillment, and it has been proved that employees perceive that their psychological contract is met when their organization deals with them through justice (Guest and Clinton, 2017). From the practical point of view, this study advocates that managers at the workplace should ensure the prevalence of justice to improve the employees’ perception regarding their psychological contract fulfillment needs so that brand-based equity should be promoted. These findings are consistent with the previous studies (Millward and Brewerton, 2000).

### Limitations and Future Directions

This study also has some potential limitations. First, this study has used only service sector employee data for the operationalization of study constructs. Thus, in the future, other study constructs should also be considered. Similarly, collecting a larger sample size in the future could be a good future avenue for the researchers. Adding respondents from multiple sectors can provide deeper insights. Adding more mediating and moderating variables, such as commitment, satisfaction, and leadership styles (Sahu et al., 2017), can provide deeper insights. Previous studies have established that the psychological contract of permanent workers is different from the psychological contract of temporary workers based on the flexible firm model (Atkinson et al., 2004) and human capital theory (Sullivan and Bhagat, 1992).

While this study has not anticipated the difference between permanent and temporary workers, thus, in the future, obtaining the perception of permanent and contractual workers can provide a different array of results, because contractual employees might face difficulties in accessing specific training (Forret and Love, 2008) that in return might limit their chance to excel and get promoted in the long run (Virtanen et al., 2003). Tenure of employment has the potency to shatter the psychological contract fulfillment (Tekleab and Taylor, 2003); thus, in the future, investigating the perception of employees having greater experience in a particular organization can provide more detailed and deeper insights regarding the fulfillment of psychological contract and brand-based equity.

## Conclusion

Based on empirical findings of this study, it can be concluded that employees perceive that their psychological contract has been met when there is a higher level of justice. This fulfillment of psychological contract further nurtures positive behaviors among the employees, and they tend to advocate positively about their organizations and promote a positive image of the organization by speaking positively about the organization. Moreover, it can also be concluded that organizations should promote the perception of justice at the workplace to trigger positive among employees. Moreover, when individuals perceive that there is a reciprocal balance based on justice, they try to find themselves in more relational obligations as feeling to repay the organizations in response to justice (Blau, 1964) and they might tend to advocate the organization under brand-based equity. Thus, balanced psychological contracts (e.g., fulfilled psychological contracts) create brand-based equity (Shore et al., 2001, 2006). Hence, psychological contracts are fulfilled when there is a match between the obligations and entitlements.

## Data Availability Statement

The original contributions presented in the study are included in the article/supplementary material, further inquiries can be directed to the corresponding author/s.

## Author Contributions

LL contributed to the work on conceptual framework, data collection and draft writing, and agreed on the final version of manuscript.

## Conflict of Interest

The author declares that the research was conducted in the absence of any commercial or financial relationships that could be construed as a potential conflict of interest.

## Publisher’s Note

All claims expressed in this article are solely those of the authors and do not necessarily represent those of their affiliated organizations, or those of the publisher, the editors and the reviewers. Any product that may be evaluated in this article, or claim that may be made by its manufacturer, is not guaranteed or endorsed by the publisher.

## References

[B1] Adams J. S. (1965). “Inequality in social exchange,” in *Advances in Experimental Social Psychology*, ed. Berkowitz L. (New York, NY: Academic Press), 267–299. 10.1016/S0065-2601(08)60108-2

[B2] Ahmad S. (2018). Can ethical leadership inhibit workplace bullying across East and West: exploring cross-cultural interactional justice as a mediating mechanism. *Eur. Manage. J.* 36 223–234. 10.1016/j.emj.2018.01.003

[B3] Akram T. Lei S. Haider M. J. Hussain S. T. Puig L. C. M. (2017). The effect of organizational justice on knowledge sharing: empirical evidence from the Chinese telecommunications sector. *J. Innov. Knowl.* 2 134–145. 10.1016/j.jik.2016.09.002

[B4] Allen A. Brady M. Robinson S. Voorhees C. (2014). One firm’s loss is another’s gain: capitalizing on other firms’ service failures. *J. Acad. Mark. Sci.* 43 648–662. 10.1007/s11747-014-0413-6

[B5] Ambler T. (2000). Marketing metrics. *Bus. Strateg. Rev.* 11 59–66. 10.1111/1467-8616.00138

[B6] Anbarasan P. Sushil (2018). Stakeholder engagement in sustainable enterprise: evolving a conceptual framework, and a case study of ITC. *Bus. Strateg. Environ.* 27 282–299. 10.1002/bse.1999

[B7] Aronsson G. Göransson S. (1999). Permanent employment but not in a preferred occupation: psychological and medical aspects, research implications. *J. Occup. Health Psychol.* 4 152–163. 10.1037//1076-8998.4.2.152 10212867

[B8] Aryee S. Budhwar P. Chen Z. (2002). Trust as a mediator of the relationship between organizational justice and work outcomes: test of a social exchange model. *J. Organ. Behav.* 23 267–285. 10.1002/job.138

[B9] Ashfaq M. Muhammad Imran Qureshi D. Irum S. Fadillah Ismail D. Rabeatul Husna D. (2018). Mediating role of psychological contract in the relationship between workplace spirituality and affective commitment. *Int. J. Eng. Technol.* 7:18335. 10.14419/ijet.v7i3.30.18335

[B10] Atkinson P. Delamont S. Coffey A. (2004). *Key Themes in Qualitative Research: Continuities and Changes.* Walnut Creek, CA: Rowman Altamira.

[B11] Badawy S. El-Fekey S. (2017). Does social comparison orientation moderate the organisational justice, in-role performance, citizenship and counterproductive behaviours relationship? *Int. J. Bus. Manage.* 12 181–193. 10.5539/ijbm.v12n12p181

[B12] Bandyopadhyay C. Srivastava K. (2021). Mediating role of psychological contract fulfilment on the relationship between strength of HR signal and organisational commitment: evidence from the Indian manufacturing sector. *S. Asian J. Hum. Resour. Manage.* 8:232209372110431. 10.1177/23220937211043109

[B13] Baumgarth C. Schmidt M. (2010). How strong is the business-to-business brand in the workforce? An empirically-tested model of ‘internal brand equity’in a business-to-business setting. *Ind. Mark. Manage.* 39 1250–1260. 10.1016/j.indmarman.2010.02.022

[B14] Beckett-Camarata J. (2003). An examination of the relationship between the municipal strategic plan and the capital budget and its effect on financial performance. *J. Public Budget. Account. Financ. Manage.* 15 23–40. 10.1108/JPBAFM-15-01-2003-B002

[B15] Bharadwaj A. (2014). Planning internal communication profile for organizational effectiveness. *IIM Kozhikode Soc. Manage. Rev.* 3 183–192. 10.1007/s11606-007-0124-5 17372791 PMC1829429

[B16] Bies R. J. (2005). “Are procedural justice and interactional justice conceptually distinct?,” in *Handbook of Organizational Justice*, eds Greenberg J. Colquitt J. A. (Hillsdale, NJ: Lawrence Erlbaum Associates Publishers), 85–112.

[B17] Blau P. (1964). *Power and Exchange in Social Life.* New York, NY: John Wiley Sons, 352.

[B18] Brethower D. M. Dickinson A. M. Johnson D. A. Johnson C. M. (2021). A history of organizational behavior management. *J. Organ. Behav. Manage.* 1–33. 10.1080/01608061.2021.1924340

[B19] Burmann C. Zeplin S. (2005). Building brand commitment: a behavioural approach to internal brand management. *J. Brand Manage.* 12 279–300. 10.1057/palgrave.bm.2540223

[B20] Burmann C. Zeplin S. Riley N. (2009). Key determinants of internal brand management success: an exploratory empirical analysis. *J. Brand Manage.* 16 264–284. 10.1057/bm.2008.6

[B21] Chandio D. Anwer D. Ashraf M. Shaikh S. (2020). Justice perception and work engagement among teachers: study of govt. business and commerce schools of Sindh. *Int. J. Psychosoc. Rehabil.* 24 10845–10852. 10.37200/IJPR/V24I7/PR271084

[B22] Christodoulides G. de Chernatony L. (2010). Consumer-based brand equity conceptualisation and measurement: a literature review. *Int. J. Mark. Res.* 52 43–66. 10.2501/S1470785310201053

[B23] Coetzee M. (2006). *The Fairness of Affirmative Action: an Organisational Justice Perspective*. Master’s thesis. Pretoria: University of Pretoria.

[B24] Collie T. Bradley G. Sparks B. A. (2002). Fair process revisited: differential effects of interactional and procedural justice in the presence of social comparison information. *J. Exp. Soc. Psychol.* 38 545–555. 10.1016/S0022-1031(02)00501-2

[B25] Connelly B. L. Certo S. T. Ireland R. D. Reutzel C. R. (2010). Signaling theory: a review and assessment. *J. Manage.* 37 39–67. 10.1177/0149206310388419

[B26] Dabos G. E. Rousseau D. M. (2004). Mutuality and reciprocity in the psychological contracts of employees and employers. *J. Appl. Psychol.* 89 52–72. 10.1037/0021-9010.89.1.52 14769120

[B27] de Chernatony L. Cottam S. (2006). Internal brand factors driving successful financial services brands. *Eur. J. Mark.* 40 611–633. 10.1108/03090560610657868

[B28] De Cremer D. (2005). Procedural and distributive justice effects moderated by organizational identification. *J. Manage. Psychol.* 20 4–13. 10.1108/02683940510571603

[B29] De Cuyper N. Baillien E. De Witte H. (2009). Job insecurity, perceived employability and targets’ and perpetrators’ experiences of workplace bullying. *Work Stress* 23 206–224. 10.1080/02678370903257578

[B30] Deepa R. Baral R. (2021). Relationship between integrated communication effectiveness and employee-based brand equity – mediating role of psychological contract fulfillment. *J. Prod. Brand Manage.* 30 883–897. 10.1108/JPBM-01-2019-2212

[B31] Deery S. J. Iverson R. D. Walsh J. T. (2006). Toward a better understanding of psychological contract breach: a study of customer service employees. *J. Appl. Psychol.* 91 166–175. 10.1037/0021-9010.91.1.166 16435946

[B32] Deluga R. J. (1994). Supervisor trust building, leader-member exchange and organizational citizenship behaviour. *J. Occup. Organ. Psychol.* 67 315–326. 10.1111/j.2044-8325.1994.tb00570.x

[B33] Dirks K. T. Ferrin D. L. (2001). The role of trust in organizational settings. *Organ. Sci.* 12 450–467. 10.1287/orsc.12.4.450.10640 19642375

[B34] Du Preez R. Bendixen M. Abratt R. (2017). The behavioral consequences of internal brand management among frontline employees. *J. Prod. Brand Manage.* 26 251–261. 10.1108/JPBM-09-2016-1325

[B35] Erkmen E. (2018). Managing your brand for employees: understanding the role of organizational processes in cultivating employee brand equity. *Adm. Sci.* 8:52. 10.3390/admsci8030052

[B36] Estreder Y. Rigotti T. Tomás I. Ramos J. (2019). Psychological contract and organizational justice: the role of normative contract. *Employee Relat. Int. J.* 42 17–34. 10.1108/ER-02-2018-0039

[B37] Festinger L. (1954). A theory of social comparison processes. *Hum. Relat.* 7 117–140. 10.1177/001872675400700202

[B38] Fornell C. Larcker D. F. (1981). Structural equation models with unobservable variables and measurement error: algebra and statistics. *J. Mark. Res.* 18, 382–388. 10.2307/3150980

[B39] Forret M. Love M. S. (2008). Employee justice perceptions and coworker relationships. *Leadersh. Organ. Dev. J.* 29 248–260. 10.1108/01437730810861308

[B40] Geisser S. (1975). The predictive sample reuse method with applications. *J. Am. Stat. Assoc.* 70 320–328. 10.1080/01621459.1975.10479865

[B41] Ghosh P. Rai A. Sinha A. (2014). Organizational justice and employee engagement: exploring the linkage in public sector banks in India. *Pers. Rev.* 43 628–652. 10.1108/PR-08-2013-0148

[B42] Goodman P. S. Haisley E. (2007). Social comparison processes in an organizational context: new directions. *Organ. Behav. Hum. Decis. Process.* 102 109–125. 10.1016/j.obhdp.2006.10.005

[B43] Greenberg J. (1987). A taxonomy of organizational justice theories. *Acad. Manage. Rev.* 12 9–22. 10.2307/257990

[B44] Greenberg J. Ashton-James C. E. Ashkanasy N. M. (2007). Social comparison processes in organizations. *Organ. Behav. Hum. Decis. Process.* 102 22–41. 10.1016/j.obhdp.2006.09.006

[B45] Griffin M. Sutton G. (2004). Integrating expectations, experiences, and psychological contract violations: a longitudinal study of new professionals. *J. Occup. Organ. Psychol.* 77 493–514. 10.1348/0963179042596487

[B46] Guerrero S. Herrbach O. (2008). The affective underpinnings of psychological contract fulfilment. *J. Manage. Psychol.* 23 4–17. 10.1108/02683940810849639

[B47] Guest D. (2004). The psychology of the employment relationship: an analysis based on the psychological contract. *Appl. Psychol.* 53 541–555. 10.1111/j.1464-0597.2004.00187.x

[B48] Guest D. E. Clinton M. (2017). “Contracting in the UK: current research evidence on the impact of flexible employment and the nature of psychological contracts,” in *Employment Contracts and Well-Being Among European Workers*, eds Cuyper N. De Isaksson K. Witte H. De (Hampshire: Ashgate), 201–223. 10.4324/9781315256573-10

[B49] Gulzar S. Ayub N. Abbas Z. (2021). Examining the mediating-moderating role of psychological contract breach and abusive supervision on employee well-being in banking sector. *Cogent Bus. Manage.* 8:1959007. 10.1080/23311975.2021.1959007

[B50] Hagtvet K. Sipos K. (2016). Creating short forms for construct measures: the role of exchangeable forms. *Pedagogika* 66 689–713.

[B51] Hair J. F. Ringle C. M. Sarstedt M. (2011). PLS-SEM: indeed a silver bullet. *J. Mark. Theory Pract.* 19 139–152. 10.2753/MTP1069-6679190202

[B52] Hair J. F. J. Hult G. T. M. Ringle C. M. Sarstedt M. (2017). *A Primer on Partial Least Squares Structural Equation Modeling (PLS-SEM).* Thousand Oaks, CA: SAGE.

[B53] Hazée S. Van Vaerenbergh Y. Armirotto V. (2017). Co-creating service recovery after service failure: the role of brand equity. *J. Bus. Res.* 74 101–109. 10.1016/j.jbusres.2017.01.014

[B54] Helm S. (2011). Employees’ awareness of their impact on corporate reputation. *J. Bus. Res.* 64 657–663. 10.1016/j.jbusres.2010.09.001

[B55] Helm S. V. Renk U. Mishra A. (2016). Exploring the impact of employees’ self-concept, brand identification and brand pride on brand citizenship behaviors. *Eur. J. Mark.* 50 58–77. 10.1108/EJM-03-2014-0162

[B56] Kashive N. Khanna V. (2017). Conceptualizing employer-based brand equity and employer brand pyramid. *Eur. Sci. J.* 13 211–229. 10.19044/esj.2017.v13n34p211

[B57] Kim T.-Y. Edwards J. R. Shapiro D. L. (2015). Social comparison and distributive justice: East Asia differences. *J. Bus. Ethics* 132 401–414. 10.1007/s10551-014-2326-1

[B58] King C. Grace D. (2010). Building and measuring employee-based brand equity. *Eur. J. Mark.* 44 938–971. 10.1108/03090561011047472

[B59] King C. Grace D. Funk D. C. (2012). Employee brand equity: scale development and validation. *J. Brand Manage.* 19 268–288. 10.1108/09526861211221518 22755484

[B60] Konovsky M. Cropanzano R. (1991). Perceived fairness of employee drug testing as a predictor of employee attitudes and job performance. *J. Appl. Psychol.* 76 698–707. 10.1037//0021-9010.76.5.698 1960142

[B61] Krejcie R. V. Morgan D. W. (1970). Determining sample size for research activities. *Educ. Psychol. Meas.* 30 607–610. 10.1177/001316447003000308

[B62] Lee J. Siu N. Zhang J. (2017). The mediating role of postrecovery satisfaction in the relationship between justice perceptions and customer attitudes. *Serv. Mark. Q.* 39 22–34. 10.1080/15332969.2017.1398025

[B63] Levinson H. Price C. R. Munden K. J. Mandl H. J. Solley C. M. (1962). *Men, Management, and Mental Health.* Cambridge, MA: Harvard University Press.

[B64] Lievens F. Van Hoye G. Anseel F. (2007). Organizational identity and employer image: towards a unifying framework. *Br. J. Manage.* 18 S45–S59. 10.1111/j.1467-8551.2007.00525.x

[B65] Malhotra N. K. Kim S. S. Patil A. (2006). Common method variance in IS research: a comparison of alternative approaches and a reanalysis of past research. *Manage. Sci.* 52 1865–1883. 10.1287/mnsc.1060.0597 19642375

[B66] Martín-de Castro G. López-Sáez P. Delgado-Verde M. (2011). Towards a knowledge-based view of firm innovation. Theory and empirical research. *J. Knowl. Manage.* 15 871–874. 10.1108/13673271111179253

[B67] McLean Parks J. Kidder D. L. Gallagher D. G. (1998). Fitting square pegs into round holes: mapping the domain of contingent work arrangements onto the psychological contract. *J. Organ. Behav.* 19 697–730. 10.1002/(sici)1099-1379(1998)19:1

[B68] Mela C. F. Kopalle P. K. (2002). The impact of collinearity on regression analysis: the asymmetric effect of negative and positive correlations. *Appl. Econ.* 34 667–677. 10.1080/00036840110058482

[B69] Millward L. J. Brewerton P. M. (2000). Psychological contracts: employee relations for the twenty-first century? *Int. Rev. Ind. Organ. Psychol.* 15 1–62.

[B70] Moon K. Lee K. Lee K. Oah S. (2016). The effects of social comparison and objective feedback on work performance across different performance levels. *J. Organ. Behav. Manage.* 37 1–12. 10.1080/01608061.2016.1236059

[B71] Moorman R. (1991). Relationship between organizational justice and organizational citizenship behaviors: do fairness perceptions influence employee citizenship? *J. Appl. Psychol.* 76 845–855. 10.1037//0021-9010.76.6.845

[B72] Morokane P. Chiba M. Kleyn N. (2016). Drivers of employee propensity to endorse their corporate brand. *J. Brand Manage.* 23 55–66. 10.1057/bm.2015.47

[B73] Morrison E. W. Robinson S. L. (1997). When employees feel betrayed: a model of how psychological contract violation develops. *Acad. Manage. Rev.* 22 226–256. 10.5465/amr.1997.9707180265

[B74] Myers C. (2003). Managing brand equity: a look at the impact of attributes. *J. Prod. Brand Manage.* 12 39–51. 10.1108/10610420310463126

[B75] Ng T. W. H. Feldman D. C. (2013). Age and innovation-related behavior: the joint moderating effects of supervisor undermining and proactive personality. *J. Organ. Behav.* 34 583–606. 10.1002/job.1802

[B76] Nimmo S. (2018). Organizational justice and the psychological contract. *Occup. Med.* 68 83–85. 10.1093/occmed/kqx115 29596673

[B77] Patterson F. (2010). Developments in work psychology: emerging issues and future trends. *J. Occup. Organ. Psychol.* 74 381–390. 10.1348/096317901167442

[B78] Piehler R. (2018). Employees’ brand understanding, brand commitment, and brand citizenship behaviour: a closer look at the relationships among construct dimensions. *J. Brand Manage.* 25 217–234. 10.1057/s41262-018-0099-z

[B79] Punjaisri K. Wilson A. (2007). The role of internal branding in the delivery of employee brand promise. *J. Brand Manage.* 15 57–70. 10.1057/palgrave.bm.2550110

[B80] Ramlall S. (2004). A review of employee motivation theories and their implications for employee retention within organizations. *J. Am. Acad. Bus.* 5 52–63.

[B81] Robinson S. L. Morrison E. W. (2000). The development of psychological contract breach and violation: a longitudinal study. *J. Organ. Behav.* 21 525–546. 10.1002/1099-1379(200008)21:5<525::AID-JOB40<3.0.CO;2-T

[B82] Saeed M. (2020). Mediation effect of psychological contract between personality dimensions and turnover intention. *J. Econ. Financ. Adm. Sci.* 25 205–219. 10.1108/JEFAS-06-2019-0101

[B83] Sahu S. Pathardikar A. Kumar A. (2017). Transformational leadership and turnover: mediating effects of employee engagement, employer branding, and psychological attachment. *Leadersh. Organ. Dev. J.* 39 82–99. 10.1108/LODJ-12-2014-0243

[B84] Saleem F. Gopinath C. (2015). Injustice, counterproductive work behavior and mediating role of work stress. *Pak. J. Commer. Soc. Sci.* 9 683–699.

[B85] Sarstedt M. Ringle C. M. Smith D. Reams R. Hair J. F. Jr. (2014). Partial least squares structural equation modeling (PLS-SEM): a useful tool for family business researchers. *J. Fam. Bus. Strateg.* 5 105–115.

[B86] Schiffman P. H. Tuncay O. C. (2001). Maxillary expansion: a meta analysis. *Clin. Orthod. Res.* 4 86–96. 10.1034/j.1600-0544.2001.040205.x 11553090

[B87] Sekiguchi T. (2007). A contingency perspective of the importance of PJ fit and PO fit in employee selection. *J. Manage. Psychol.* 22 118–131. 10.1108/02683940710726384

[B88] Shinnar R. S. Young C. A. Meana M. (2004). The motivations for and outcomes of employee referrals. *J. Bus. Psychol.* 19 271–283. 10.1007/s10869-004-0552-8

[B89] Shore L. M. Tetrick L. E. Barksdale K. (2001). *Social and Economic Exchanges as Mediators of Commitment and Performance*. Unpubl. Manuscr.

[B90] Shore L. M. Tetrick L. E. Lynch P. Barksdale K. (2006). Social and economic exchange: construct development and validation. *J. Appl. Soc. Psychol.* 36 837–867. 10.1111/j.0021-9029.2006.00046.x

[B91] Sirianni N. J. Bitner M. J. Brown S. W. Mandel N. (2013). Branded service encounters: strategically aligning employee behavior with the brand positioning. *J. Mark.* 77 108–123. 10.1509/jm.11.0485 11670861

[B92] Snell S. A. Dean J. W. (1992). Integrated manufacturing and human resource management: a human capital perspective. *Acad. Manage. J.* 35 467–504. 10.2307/256484

[B93] Spence J. Ferris D. Brown D. Heller D. (2011). Understanding daily citizenship behaviors: a social comparison perspective. *J. Organ. Behav.* 32 547–571. 10.1002/job.738

[B94] Sturges J. Conway N. Guest D. Liefooghe A. (2005). Managing the career deal: the psychological contract as a framework for understanding career management, organizational commitment and work behavior. *J. Organ. Behav.* 26 821–838. 10.1002/job.341

[B95] Suliman A. Al Kathairi M. (2013). Organizational justice, commitment and performance in developing countries. *Employee Relat.* 35 98–115. 10.1108/01425451311279438

[B96] Sullivan S. E. Bhagat R. S. (1992). Organizational stress, job satisfaction and job performance: where do we go from here? *J. Manage.* 18 353–374. 10.1177/014920639201800207

[B97] Tekleab A. G. Taylor M. S. (2003). Aren’t there two parties in an employment relationship? Antecedents and consequences of organization–employee agreement on contract obligations and violations. *J. Organ. Behav.* 24 585–608. 10.1002/job.204

[B98] Turnley W. H. Bolino M. C. Lester S. W. Bloodgood J. M. (2003). The impact of psychological contract fulfillment on the performance of in-role and organizational citizenship behaviors. *J. Manage.* 29 187–206. 10.1016/S0149-2063(02)00214-3

[B99] Van Yperen N. W. Brenninkmeijer V. Buunk A. P. (2006). People’s responses to upward and downward social comparisons: the role of the individual’s effort-performance expectancy. *Br. J. Soc. Psychol.* 45(Pt 3) 519–533. 10.1348/014466605X53479 16984718

[B100] Veloutsou C. Guzman F. (2017). The evolution of brand management thinking over the last 25 years as recorded in the. *J. Prod. Brand Manage.* 26 2–12. 10.1108/JPBM-01-2017-1398

[B101] Virtanen M. Kivimäki M. Virtanen P. Elovainio M. Vahtera J. (2003). Disparity in occupational training and career planning between contingent and permanent employees. *Eur. J. Work Organ. Psychol.* 12 19–36. 10.1080/13594320344000002

[B102] Zhao L. Lu Y. Zhang L. Chau P. Y. K. (2012). Assessing the effects of service quality and justice on customer satisfaction and the continuance intention of mobile value-added services: an empirical test of a multidimensional model. *Decis. Support Syst.* 52 645–656. 10.1016/j.dss.2011.10.022

